# Medical laboratory science course performance and grade point average as indicators of ASCP certification success: a 10-year review from an NAACLS-accredited program

**DOI:** 10.1093/labmed/lmag024

**Published:** 2026-06-18

**Authors:** Sumbul Bushra, Rana Abozoor, Taghreed Abu-Nada, Mohammed Al-Hamdani, Atiyeh M Abdallah, Marawan Abu-Madi

**Affiliations:** Department of Biomedical Sciences, College of Health Sciences, QU Health, Qatar University, Doha, Qatar; Department of Public Health, College of Health Sciences, QU Health, Qatar University, Doha, Qatar; Department of Biomedical Sciences, College of Health Sciences, QU Health, Qatar University, Doha, Qatar; Department of Public Health, College of Health Sciences, QU Health, Qatar University, Doha, Qatar; Department of Biomedical Sciences, College of Health Sciences, QU Health, Qatar University, Doha, Qatar; Department of Biomedical Sciences, College of Health Sciences, QU Health, Qatar University, Doha, Qatar

**Keywords:** medical laboratory science, GPA, ASCP certification, NAACLS-accredited, performance, Qatar University

## Abstract

**Introduction:**

Passing the American Society for Clinical Pathology Board of Certification (ASCP BOC) exam is a key milestone, and this study examined its association with academic performance at Qatar University.

**Methods:**

For 228 graduates attempting the ASCP BOC exam, use used χ^2^ tests to assess relationships between (1) performance in ASCP domains and overall exam result (pass/fail) and (2) course grades (C or above vs below C) and ASCP BOC domain performance. The correlation between grade point average (GPA) and ASCP BOC exam score was assessed using the Pearson coefficient.

**Results:**

Passing any ASCP BOC domain was statistically significantly associated with passing the overall exam (all *P* < .001). Students who passed the overall exam demonstrated high pass rates across domains, particularly in chemistry (95.5%), hematology (84.1%), microbiology (86.0%), and blood banking (73.9%). Achieving a grade of C or higher in courses at the university was statistically significantly associated with passing the corresponding ASCP BOC domains in most cases. Graduation GPA was positively correlated with total ASCP BOC score (*r* = 0.652, *P* < .001).

**Discussion:**

Academic performance at the university level is strongly associated with ASCP BOC certification success, along with other factors that influence student success. Higher graduation GPA and passing grades in core courses are strong predictors of improved ASCP BOC exam outcomes, providing early indicators by which to identify students who may benefit from targeted academic support.

## Introduction

Dedicated to the enhancement of education in clinical laboratory sciences and health care disciplines, the National Accrediting Agency for Clinical Laboratory Sciences (NAACLS) provides accreditation to domestic and international medical laboratory sciences (MLS) programs.^1^^,^^2^ Accredited by the NAACLS, the mission of the MLS program at the Biomedical Science Department, College of Health Sciences, Qatar University (QU), is to prepare competent biomedical scientists who value quality, value, and patient safety. As an NAACLS-accredited program, the QU Biomedical Science program seeks to train graduates who possess not only the requisite knowledge, skills, and values but also who can execute these skills in clinical laboratory practice.

NAACLS accreditation helps graduates attain the American Society for Clinical Pathology Board of Certification (ASCP BOC) examination. The use of computer adaptive testing in the ASCP BOC exam increases the difficulty of the questions with every correct answer and decreases the difficulty with every incorrect answer. Examinees require a score of 400 out of a possible 999 to achieve certification.^3^ An average ASCP BOC pass rate of 75% over 3 consecutive years for graduates attempting the exam within the first year of graduation is 1 of the obligatory criteria the NAACLS monitors when assessing the accreditation status of MLS programs.^4^ This accreditation requirement means that it is essential to assess the relationship between academic performance in the MLS program and examination performance to evaluate curriculum efficacy and student readiness.^5^ Passing certification exams tends to be an indicator of advanced and expert competencies, so licensed graduates are preferred in the workplace.^6^

Globally, certification examinations such as the ASCP BOC serve as a benchmark to ensure that graduates entering the clinical laboratory workforce meet standardized competencies in analytical reasoning, quality assurance, and patient-centered laboratory practice. As health care systems expand and laboratory diagnostics become increasingly complex, the demand for highly skilled and credentialed laboratory professionals continues to rise. Many employers now prefer or mandate ASCP certification as an indicator of readiness for independent professional practice, reinforcing the importance of strong academic foundations in MLS programs.^7^^,^^8^ For countries in the Middle East, including Qatar, strengthening certification outcomes is essential to supporting national health care strategies, improving diagnostic capacity, and aligning workforce competencies with international best practices.

Given this growing emphasis on certification-based readiness, understanding which academic factors are associated with success on the ASCP BOC examination has important implications for curriculum planning, academic advising, and student support. Increasingly, MLS programs rely on data-driven approaches to identify students who may be at risk of underperforming and to implement targeted interventions early in their academic journey.^9^ For QU, where the student population is diverse and often includes first-generation university students, identifying clear academic indicators of certification performance can help tailor instructional strategies, enhance course alignment with professional competencies, and ultimately strengthen graduate employability. Despite the global relevance of certification predictors, research into these factors is limited in the context of MLS programs outside North America, making it essential to explore how local academic structures and learning environments relate to ASCP BOC outcomes.

Nevertheless, studies of predictors of ASCP BOC outcomes have been conducted. Some of these studies have reported that mode of instruction but not preadmission grade point averages (GPAs) or years to graduation was positively associated with passing the ASCP BOC examination.^10^ In another study, the total admission score and nonscience and science GPA were moderately but statistically significantly associated with ASCP BOC examination score.^11^ Previous studies have suggested that performance on comprehensive university examinations may help predict student outcomes on the ASCP BOC examination, supporting efforts to optimize curriculum design and provide targeted academic support. Pelton et al^12^ detected an overall moderate correlation between overall university scores and ASCP BOC examination scores but poor correlations with individual subjects, with a cutoff of 60% in university examinations predicting ASCP BOC success. In another, similar study, a cutoff of approximately 75% in the university senior comprehensive examination predicted ASCP BOC success.^13^ The length of time between graduation and taking the ASCP BOC examination also appears to be associated with examination outcome, with a statistically significant increase in the failure rate from 8.9% in graduates attempting the examination within 1 to 3 months after graduation to 26.4%, 30.4%, and 31.9% for individuals attempting it 4 to 6 months, 7 to 9 months, and 10 to 12 months after program completion, respectively.^14^ Several studies have explored GPA as a strong predictor of both academic and certification examination success, with Thomas and Alexander^15^ reporting a statistically significant positive association between preadmission GPA and ASCP BOC performance in MLS graduates.  Hubbard and Sawyer^11^ found that although academic measures such as GPA and total admission score were substantial positive predictors of student success within a clinical laboratory program, GPA alone was not a strong predictor of performance on the ASCP BOC examination.

There is still a need to identify factors associated with ASCP BOC examination outcomes, however, especially those that might allow for early intervention and targeted educational support.^16^ Therefore, the objective of this study was to examine the association between university-based academic metrics (rather than preadmission criteria^12^), such as GPA and individual course performance, and ASCP BOC examination outcomes in college students pursuing a biomedical science degree at QU. To reinforce the transferability of these educational fundamentals to varied cultural and educational settings, we explore relationships between academic performance and certification success in the context of a non–North American environment, an international NAACLS-accredited program in Qatar. In doing so, this study facilitated a deeper understanding of the efficacy of current pedagogical strategies in promoting the requisite knowledge and skills for professional practice and to align university-level instruction and teaching with the competencies required by certifying bodies and the ASCP-BOC.^17–20^ It also suggested that the correlation between an evidence-based, accredited curriculum and professional readiness persists regardless of geographical, jurisdictional, or institutional context. Our findings offer guidance to an increasing number of international MLS programs undergoing NAACLS accreditation and seeking to prepare graduates for certification internationally.

## Methods

### Research design

This retrospective, quantitative, cross-sectional study focused on the relationship between academic performance in the bachelor of science in biomedical sciences (MLS program) at QU and graduate performance on the ASCP BOC examination. The Institutional Review Board of QU approved the study (QU-IRB 1360-EA/20). All procedures conformed to QU’s research ethics policies and NAACLS confidentiality requirements. No identifiable student information was disclosed. The study used secondary institutional data; therefore, informed consent was waived, consistent with ethical guidelines for retrospective academic performance research.

### Setting and participants

This study was conducted at the Department of Biomedical Sciences, College of Health Sciences, QU, an NAACLS-accredited program. Data from 228 MLS graduates who completed the program and attempted the ASCP BOC examination between 2018 and 2024 were analyzed. The inclusion criteria were successful completion of all didactic and clinical requirements in the MLS curriculum; first attempt at the ASCP BOC examination within 1 year of graduation; and availability of complete academic records (course grades, cumulative GPA, and ASCP BOC examination results). Students who did not attempt the ASCP BOC examination or who attempted it more than 1 year after graduation were excluded to minimize time effects on outcomes.

### Data sources and variables

Archival data were obtained from the departmental records and verified through the Office of the Registrar. The primary outcome was ASCP BOC examination outcome. Correlates included graduation GPA (continuous on a 0-4 scale); course-specific performance (chemistry, hematology, microbiology, blood banking, immunology/serology, urinalysis, and laboratory operations, dichotomized as a grade of at least a C or below a C); and demographic variables (sex, nationality, year of graduation).

### Data collection

Deidentified graduate data were extracted, coded, and entered into Microsoft Excel before statistical analysis. Each record included a unique numeric identifier to ensure confidentiality. No personal identifiers were used.

### Statistical analysis

We used χ^2^ tests to evaluate (1) the relationship between performance in ASCP BOC examination domains (pass or fail) and on the overall ASCP BOC examination (pass or fail) and (2) the relationship between grade threshold (at least a C or below a C) in QU subjects and overall or individual subject ASCP BOC examination performance. We calculated the Pearson correlation coefficients to analyze the correlation between the overall ASCP BOC examination score and graduation GPAs.

Course performance was dichotomized as a grade below C vs at least a C a priori because a grade of C represents the program’s established minimum competency benchmark and the operational threshold used in academic progression decisions. In routine advising, a grade below C triggers structured remediation and enhanced academic support, making this cut point directly actionable for early identification of students at risk. Rather than optimizing a cohort-specific data-driven cutoff, which would have limited generalizability, selecting a grade of at least a C as the threshold therefore preserves interpretability and alignment with real-world educational practices.

## Results

Overall ASCP BOC examination performance (pass/fail) was strongly associated with performance in each ASCP BOC domain examination (pass/fail): blood banking (immunohematology), 73.9%; urinalysis and other body fluids, 79.6%; chemistry, 95.5%; hematology, 84.1%; immunology/serology, 86%; microbiology, 86%; and laboratory operations, 68.8% (Table 1). Individuals who passed all the ASCP BOC domains were much more likely to pass the overall ASCP BOC examinations, with the association highest for hematology (difference, 67.1%) and lowest for laboratory operations (difference, 40.6%) (Table 1).

**Table 1:** Association between ASCP BOC examination domain performance and overall ASCP BOC examination performance (*n* = 228).

ASCP domain performance		*df*	χ^2^	*P* value	Overall ASCP BOC examination performance			
					Failed ASCP BOC, No. (%)	Passed ASCP BOC, No. (%)	Difference in fail vs pass, %	Overall, No. (%)
Blood banking (immunohematology)	Fail	1	73.9	<.001	62 (87.3)	41 (26.1)	61.2	103 (45.2)
	Pass				9 (12.7)	116 (73.9)		125 (54.8)
Urinalysis and other body fluids	Fail	1	76.9	<.001	58 (81.7)	32 (20.4)	61.3	90 (39.5)
	Pass				13 (18.3)	125 (79.6)		138 (60.5)
Chemistry	Fail	1	113.4	<.001	50 (70.4)	7 (4.5)	65.9	57 (25)
	Pass				21 (29.6)	150 (95.5)		171 (75)
Hematology	Fail	1	94.8	<.001	59 (83.1)	25 (15.9)	67.1	84 (36.8)
	Pass				12 (16.9)	132 (84.1)		144 (63.2)
Immunology/serology	Fail	1	60.2	<.001	46 (64.8)	22 (14)	50.7	68 (29.8)
	Pass				25 (35.2)	135 (86)		160 (70.2)
Microbiology	Fail	1	51.9	<.001	43 (60.6)	22 (14)	46.5	65 (28.5)
	Pass				28 (39.4)	135 (86)		163 (71.5)
Laboratory operations	Fail	1	32.7	<.001	51 (71.8)	49 (31.2)	40.6	100 (43.9)
	Pass				20 (28.2)	108 (68.8)		128 (56.1)

Abbreviation: ASPC BOC, American Society for Clinical Pathology Board of Certification.

Associations between grade threshold (C = 70% or above vs below a C) for QU courses and performance in ASCP BOC domains are shown in Table 2. For most of the evaluated courses, there were substantial associations between the grade achieved (C = 70% or above vs below a C) in QU courses and performance in the respective ASCP BOC domains (pass or fail). In particular, a greater proportion of individuals passed than failed the overall ASCP BOC examination for those scoring above a grade of C for the following QU courses: microbiology (85.3%), chemistry (90.6%), hematology (92.4%), urinalysis and other body fluids (81.9%), and blood banking (95.2%). There was no association between passing or failing the immunology/serology or laboratory operations sections of the ASCP BOC examination and the respective QU courses (*P* = .08 and *P* = .41, respectively). The difference in the proportion of overall ASCP BOC examination passes and fails was largest for test-takers who scored above a C in hematology (difference, 19.7%) and lowest for those scoring above a C in laboratory operations (difference, 2.4%).

**Table 2:** Association between grade threshold in QU courses and performance in the corresponding ASCP BOC domains (*n* = 228).

University subject		*df*	χ^2^	*P* value	Corresponding ASCP BOC domain			
					Failed ASCP BOC, No. (%)	Passed ASCP BOC, No. (%)	Difference in fail vs pass, %	Overall, No. (%)
Blood banking (immunohematology)	Grade (≥C)	1	5.4	.03	89 (86.4)	119 (95.2)	8.8	208 (91.2)
	Grade (<C)				14 (13.6)	6 (4.8)		20 (8.8)
Urinalysis and other body fluids	Grade (≥C)	1	9.8	.003	57 (63.3)	113 (81.9)	18.5	170 (74.6)
	Grade (<C)				33 (36.7)	25 (18.1)		58 (25.4)
Chemistry	Grade (≥C)	1	12.4	.001	41 (71.9)	155 (90.6)	18.7	196 (86)
	Grade (<C)				16 (28.1)	16 (9.4)		32 (14)
Hematology	Grade (≥C)	1	16.2	<.001	61 (72.6)	133 (92.4)	19.7	194 (85.1)
	Grade (<C)				23 (27.4)	11 (7.6)		34 (14.9)
Immunology/serology	Grade (≥C)	1	3.5	.08	55 (80.9)	144 (90)	9.1	199 (87.3)
	Grade (<C)				13 (19.1)	16 (10)		29 (12.7)
Microbiology	Grade (≥C)	1	6.3	.015	46 (70.8)	139 (85.3)	14.5	185 (81.1)
	Grade (<C)				19 (29.2)	24 (14.7)		43 (18.7)
Laboratory operations	Grade (≥C)	1	1.3	.41	96 (96)	126 (98.4)	2.4	222 (97.4)
	Grade (<C)				4 (4)	2 (1.6)		6 (2.6)

Abbreviations: ASPC BOC, American Society for Clinical Pathology Board of Certification; QU, Qatar University.

The association between grade threshold for QU courses and overall ASCP BOC examination performance is shown in Table 3. For most of the courses evaluated, there were statistically significant associations between QU grade and overall ASCP BOC examination performance (pass or fail). In particular, a greater proportion of individuals passed than failed the ASCP BOC examination for those who scored above a C in microbiology (89.1%), chemistry (93.6%), hematology (91.7%), urinalysis and other body fluids (86.0%), immunology/serology (93.6%), and blood banking (95.5%) but not laboratory operations (*P* = .38). The difference in the proportion of overall ASCP BOC examination passes vs failures was largest for individuals scoring above a C in urinalysis and other body fluids (difference, 36.7%) and lowest for those who scored above a C in laboratory operations (difference, 2.3%).

**Table 3:** Association between grade threshold in QU courses and overall ASCP BOC examination performance (*n* = 228).

University subject		*df*	χ^2^	*P* value	Overall ASCP BOC examination performance			
					Failed ASCP BOC, No. (%)	Passed ASCP BOC, No. (%)	Difference in fail vs pass, %	Overall, No. (%)
Blood banking (immunohematology)	Grade (≥C)	1	11.7	.002	58 (81.7)	150 (95.5)	13.8	208 (91.2)
	Grade (<C)				13 (18.3)	7 (4.5)		20 (8.8)
Urinalysis and other body fluids	Grade (≥C)	1	34.7	<.001	35 (49.3)	135 (86.0)	36.7	170 (74.6)
	Grade (<C)				36 (50.7)	22 (14.0)		58 (25.4)
Chemistry	Grade (≥C)	1	24.6	<.001	49 (69.0)	147 (93.6)	24.6	196 (86)
	Grade (<C)				22 (31.0)	10 (6.4)		32 (14)
Hematology	Grade (≥C)	1	17.5	<.001	50 (70.4)	144 (91.7)	21.3	194 (85.1)
	Grade (<C)				21 (29.6)	13 (8.3)		34 (14.9)
Immunology/serology	Grade (≥C)	1	18.3	<.001	52 (73.2)	147 (93.6)	20.4	199 (87.3)
	Grade (<C)				19 (26.8)	10 (6.4)		29 (12.7)
Microbiology	Grade (≥C)	1	21.3	<.001	45 (63.4)	140 (89.1)	25.8	185 (81.1)
	Grade (<C)				26 (36.6)	17 (10.8)		43 (18.7)
Laboratory operations	Grade (≥C)	1	1.0	.38	68 (95.8)	154 (98.1)	2.3	222 (97.4)
	Grade (<C)				3 (4.2)	3 (1.9)		6 (2.6)

Abbreviations: ASPC BOC, American Society for Clinical Pathology Board of Certification; QU, Qatar University.

**Figure 1:**
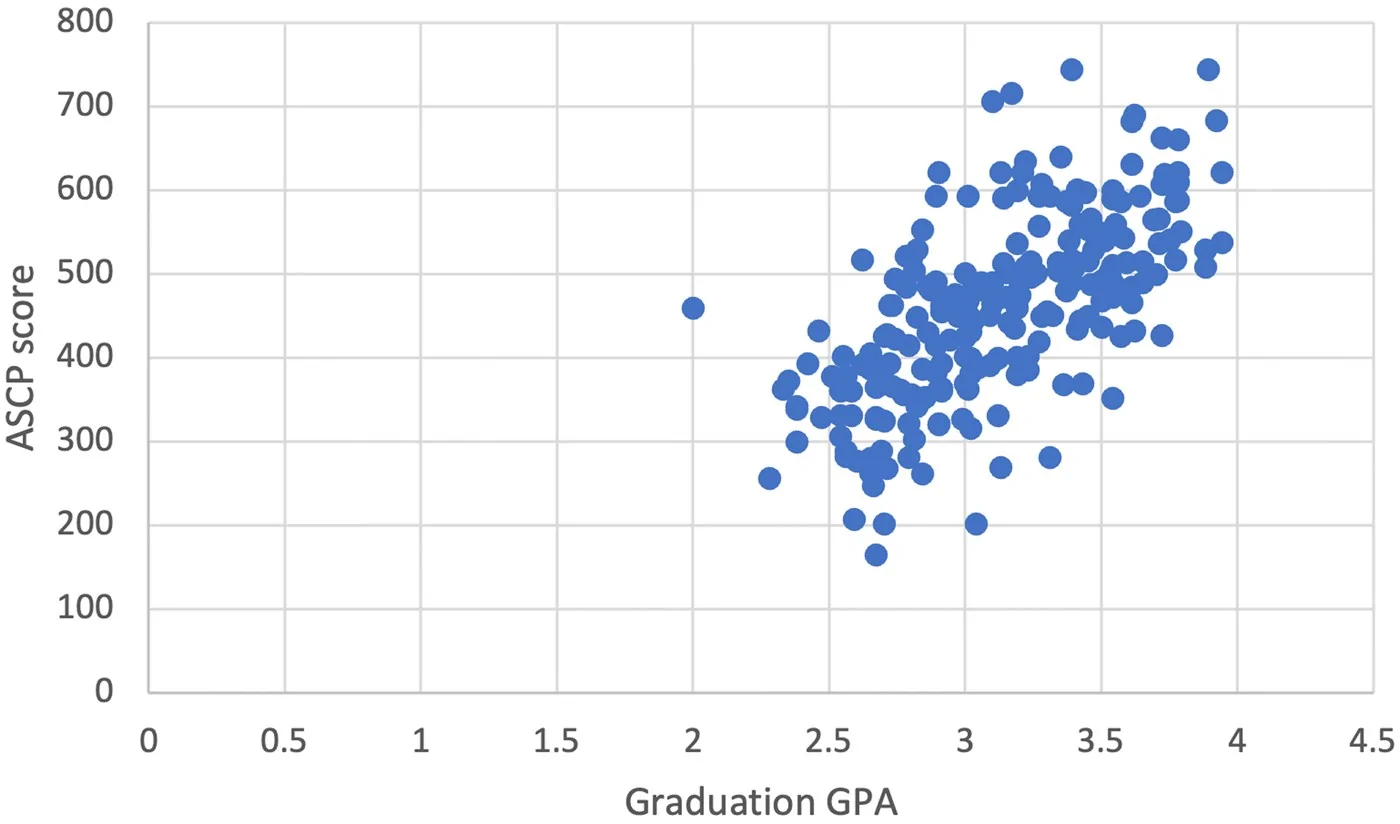
Plot showing the correlation between grade point average (GPA) at graduation and overall American Society for Clinical Pathology Board of Certification (ASCP BOC) examination score (*r* (*n* = 228)  = 0.652, *P* < .001).

A higher GPA was correlated with better ASCP BOC examination scores, as evidenced by the statistically significant positive correlation between GPA at graduation and overall ASCP BOC examination score (*r* (*n* = 228) = 0.652; *P* < .001) (Figure 1).

## Discussion

This study examined (1) the relationship between ASCP BOC examination performance in individual domains and overall, (2) the relationship between grade achieved in QU courses and examination performance in the corresponding ASCP BOC domains, and (3) the correlation between graduation GPAs and overall ASCP BOC examination scores. Passing (vs failing) any of the ASCP BOC domains was strongly associated with passing the overall exam. In addition, achieving a grade of C or above in QU courses was associated with a higher likelihood of passing the corresponding ASCP BOC domain examination for at least 5 of the 7 domains and with passing the ASCP BOC overall examination for at least 6 of the 7 domains. Finally, graduation GPAs were positively correlated with overall ASCP BOC examination scores.

One of the study’s main outcomes was that passing any of the ASCP BOC domain examinations was strongly correlated with passing the ASCP BOC examination overall, highlighting the importance of competency in each domain for overall success. Previous studies have shown a strong correlation between performance in certain ASCP BOC examination domains and overall examination success. Pelton et al^12^ reported a perfect correlation (*r* = 1.000) between passing the ASCP BOC examination and comprehensive examination scores above 74.36%, with a focus on a single, preinternship comprehensive exam. Instead, the current study measured the performance variables at the point of graduation. This data collection timing reflects the cumulative knowledge and skills acquired throughout the entire MLS program, including all didactic and clinical internships. Therefore, this metric assesses the effectiveness of the complete educational experience in preparing students for certification. The significance of domain-specific abilities in certification outcomes was further demonstrated in the American Society for Clinical Laboratory Science study,^21^ which revealed that students with high affective scores were more likely to pass the MLS BOC examination on their first try. This result highlights the importance of focused preparation and assessment in MLS education and the need for courses that prioritize expertise in specific laboratory science fields to improve examination results. Educational programs should focus assessment and preparedness efforts on individual domains where student performance is weaker to enhance applicant readiness and competence. Ultimately, this focused approach to training and evaluation can improve the standard of laboratory experts who join clinical practice.

The greatest differences in the proportion of failures vs passes for overall performance on the ASCP BOC examination were for hematology, chemistry, urinalysis, and blood banking; the least difference was for laboratory operations. This finding suggests that laboratory operations courses have the least impact on overall ASCP BOC examination performance, although this may be because this domain contains fewer test items. Future initiatives should focus on continuing to support students in hematology, chemistry, urinalysis, and blood banking given the clear association between passing these domains and overall examination performance, perhaps through extra workshops for examination preparation.

A second key finding of this study was that achieving a grade of C or higher in QU subjects was associated with a higher likelihood of passing the corresponding ASCP BOC domain examinations, except for laboratory operations and immunology/serology, where most scored a grade of C or above. This finding is consistent with a previous study indicating a direct correlation between subject-specific performance and national certification outcomes^13^; students who scored higher than approximately 74% in their senior departmental comprehensive examination were much more likely to pass the Medical Technologist ASCP BOC certification examination on their first try. This finding suggests that the QU curriculum effectively prepares students for the ASCP BOC examination and that the professional skills tested through the ASCP BOC examination are strongly aligned with existing coursework. It also indicates that a threshold of a grade of C or above could be a target in terms of student performance. This approach allows for a more granular understanding of how performance in specific curricular domains, such as chemistry, hematology, and microbiology, translates to success in the corresponding sections of the ASCP BOC examination. By using dichotomized course grades (a grade of C or higher vs below a C), the current study established a practical, easily identifiable benchmark for student performance integrated into the existing academic structure that does not require additional, specialized testing. This approach allows for early and continuous identification of at-risk students throughout their academic journey.

A third key finding in this study was that most students who scored a grade of C or above in a university subject corresponding to an ASCP BOC domain were more likely to pass that specific domain and the overall ASCP BOC examination in most of the 7 domains. Importantly, performance in the urinalysis and other body fluids, chemistry, and microbiology courses had the greatest impact on ASCP BOC examination success, suggesting that obtaining at least a grade of C is particularly important for these courses. It also suggests that these courses prepare students well for passing the examination.

Finally, we detected a statistically significant positive correlation between GPA at graduation and overall ASCP BOC examination score, indicating that higher academic achievement during university is associated with subsequent examination success. This result is consistent with the American Society for Clinical Laboratory Science study, which showed a statistically significant correlation between passing the ASCP BOC examination and a higher GPA (and outstanding course performance).^21^ These results emphasize how course grades and cognitive academic achievement are indicators of certification examination success. The GPA might be useful as an early screening tool to find students who are likely to perform poorly subsequently, providing an opportunity for provision of focused interventions, such as academic advice, extra instruction, or organized review sessions. Furthermore, early detection of at-risk students through their GPA provides students with the opportunity to improve their readiness by addressing their failures early through formative assessments and mock examinations. Together, improving academic performance at college, especially by maintaining a GPA over the cutoff associated with certification achievement, might increase students’ preparedness for the ASCP BOC examination and increase pass rates. The GPA continues to be 1 of the most important indicators of strong academic performance.

It is important to note that not all students who achieved a grade of C or higher in core courses passed the corresponding ASCP BOC domain examinations or the overall examination, reflecting the multifaceted nature of success in certification examinations. Several non–mutually exclusive factors contribute to certification success. First, university courses typically conduct multiple assessments and examinations over a semester that contribute to individual student grades. This type of assessment may not necessarily translate into success in a single, comprehensive, computer-adaptive examination that demands both content mastery and sophisticated test-taking skills, knowledge integration across domains, and the ability to achieve under time pressure and psychological stress. Second, there is a considerable time gap between completing foundational courses early in the academic program and the certification examination, which could lead to knowledge retention and decay. Third, study techniques suited to success in individual courses may not be suitable for certification examinations. Finally, noncognitive factors such as test anxiety, inadequate examination preparation due to overconfidence, or personal circumstances can substantially affect performance in otherwise knowledgeable and capable students. Our findings highlight that although course grade is a statistically significant predictor of certification success, it is not deterministic of success. Academic measurements and predictors should be considered useful tools for identifying students who may benefit from added support, bearing in mind that a holistic approach to examination preparation, including development of test-taking skills, cumulative review techniques, and addressing affective factors, is necessary to optimize outcomes.

The results of this study also provide practical guidance for curriculum evaluation and student support within MLS programs. The strong associations between subject performance, GPA, and ASCP BOC examination outcomes suggest that several core QU courses, particularly chemistry, hematology, microbiology, urinalysis, and blood banking, serve as meaningful indicators of certification readiness. These findings can help faculty identify where reinforcement is most needed, whether through revised course mapping, increased emphasis on competencies outlined by ASCP BOC, or more structured review opportunities in courses with high predictive value. Conversely, the weaker associations observed for immunology/serology highlight areas where instructional strategies, assessment design, or competency alignment may benefit from re-examination. In parallel, the relationship between GPA and ASCP BOC examination success supports the use of academic performance as an early advising tool to guide examination readiness decisions and targeted academic interventions. These insights can support the overall preparation of students entering professional practice.

This study has several limitations. The data used in this study were from a single institution, which may limit generalizability to other universities or areas with different student populations and curricula. Several potentially substantial factors that could have influenced examination outcomes were not evaluated, such as study habits, motivation, affective performance (professional attitudes and behaviors), and previous clinical or work experience. Furthermore, the study focused primarily on quantitative measures, including grades, GPA, and examination performance, overlooking qualitative perspectives from instructors or students about preparation challenges or support strategies. In addition, the study did not account for other family support measures or access to extracurricular educational resources that might help students improve their performance. It is important to recognize that a variety of factors contribute to student performance in this high-stakes examination. For example, Doxtater and Cruz^21^ found that MLS students with high affective scores for self-efficacy, academic compliance, and the ability to manage negative environmental factors had a 94.7% pass rate compared with a 57.1% pass rate for students with low affective scores. A systematic review reported that the use of mock or practice examinations, including those that address the timing and format of resources, are a positive factor in examination success in various health care professions.^22^ Examination performance may also be influenced by test-taking anxiety because anxious students are reported to have lower academic motivation, creating a cycle of anxiety and poor performance.^23^ The same study also demonstrated that students who can effectively manage their time feel more in control of their studies, in turn boosting their motivation, reducing anxiety, and enhancing their examination performances.^23^ Improving student success on the ASCP BOC examination requires a holistic approach that addresses not only cognitive learning but also the affective and behavioral aspects of student development.

Overall, the study reveals that academic performance, including achieving passing grades in relevant courses and a higher GPA, is associated with ASCP BOC examination success. These findings suggest that academic measures, including GPA, are early indicators by which to identify students who may be in need of targeted support and interventions. Although the study has limitations, the results provide valuable perspectives for curriculum planning and student preparation strategies. Finally, fostering academic success during university can improve students’ readiness and certification outcomes.

## Data Availability

The data supporting the findings of this study are available from the corresponding author upon reasonable request.
