# Correction to: Medical laboratory science course performance and grade point average as indicators of ASCP certification success: a 10-year review from an NAACLS-accredited program

**DOI:** 10.1093/labmed/lmag066

**Published:** 2026-09-12

This is a correction to: Sumbul Bushra, Rana Abozoor, Taghreed Abu-Nada, Mohammed Al-Hamdani, Atiyeh M Abdallah, Marawan Abu-Madi, Medical laboratory science course performance and grade point average as indicators of ASCP certification success: a 10-year review from an NAACLS-accredited program, *Laboratory Medicine*, Volume 57, Issue 4, July 2026, lmag024, https://doi.org/10.1093/labmed/lmag024

In the originally published version of this manuscript, there was a typographical error in the fourth co-author’s name.

This has been corrected to read: “Mohammed Al-Hamdani.”

